# “Life is short, and art is long”: RNA degradation in cyanobacteria and model bacteria

**DOI:** 10.1002/mlf2.12015

**Published:** 2022-03-24

**Authors:** Ju‐Yuan Zhang, Wolfgang R. Hess, Cheng‐Cai Zhang

**Affiliations:** ^1^ State Key Laboratory of Freshwater Ecology and Biotechnology and Key Laboratory of Algal Biology, Institute of Hydrobiology Chinese Academy of Sciences Wuhan China; ^2^ Genetics and Experimental Bioinformatics, Faculty of Biology University of Freiburg Freiburg Germany; ^3^ Institut WUT‐AMU Aix‐Marseille University and Wuhan University of Technology Wuhan China

**Keywords:** cyanobacteria, RNA maturation, RNA metabolism, RNA turnover, ribonucleases

## Abstract

RNA turnover plays critical roles in the regulation of gene expression and allows cells to respond rapidly to environmental changes. In bacteria, the mechanisms of RNA turnover have been extensively studied in the models *Escherichia coli* and *Bacillus subtilis*, but not much is known in other bacteria. Cyanobacteria are a diverse group of photosynthetic organisms that have great potential for the sustainable production of valuable products using CO_2_ and solar energy. A better understanding of the regulation of RNA decay is important for both basic and applied studies of cyanobacteria. Genomic analysis shows that cyanobacteria have more than 10 ribonucleases and related proteins in common with *E. coli* and *B. subtilis,* and only a limited number of them have been experimentally investigated. In this review, we summarize the current knowledge about these RNA‐turnover‐related proteins in cyanobacteria. Although many of them are biochemically similar to their counterparts in *E. coli* and *B. subtilis,* they appear to have distinct cellular functions, suggesting a different mechanism of RNA turnover regulation in cyanobacteria. The identification of new players involved in the regulation of RNA turnover and the elucidation of their biological functions are among the future challenges in this field.

## INTRODUCTION

### “Life is short, and art is long”—Hippocrates

Gene expression is regulated at multiple levels, and the stability of RNA is a key regulatory step for gene expression. As intermediates of genetic information decoding process, the relative amounts of RNAs reflect the balance between transcription and degradation. Compared to proteins, the half‐lives of RNAs, in particular, those of messenger RNAs (mRNAs), are very short. In *Escherichia coli*, for example, the half‐life of mRNA averages 6.8 min, ranging from less than a minute to more than 10 min^1^, ^2^, while a median half‐life of 2.4 min was determined for mRNAs from the cyanobacterium *Prochlorococcus* sp. MED4^3^. The rapid turnover of RNA constitutes an important mechanism of gene regulation and enables a cell to respond in a timely manner to changes in the environment. Beyond their role as carriers of genetic information, RNAs can be a structural component, such as the rRNAs in the ribosome; function as guide RNAs such as CRISPR RNAs (crRNAs); or have regulatory functions, such as riboswitches, trans‐acting small RNAs (sRNAs), or antisense RNAs. Therefore, studying the process of RNA decay is essential for our general understanding of gene regulation.

Bacterial RNA degradation has been extensively studied in the gram‐negative model *E. coli*, and to a lesser extent, in the gram‐positive model *Bacillus subtilis*. RNA decay requires a large number of proteins, which can be categorized into three groups: endoribonucleases, exoribonucleases, and auxiliary proteins^4^, ^5^ (Table 1). In *E. coli*, the endoribonucleases include RNase E, which cleaves internally in most RNA species; RNases Z and P, which generate the mature ends of transfer RNAs (tRNAs); RNase III, which cleaves at specific sites within double‐stranded RNAs (dsRNAs); and RNase H, which cuts the RNA strand in RNA–DNA duplexes. The exoribonucleases include PNPase, RNase R, and RNase II, which contribute to the degradation of bulk RNA; RNase D and PH, which mainly act on tRNA precursors; and Orn, which specifically degrades oligoribonucleotides. All these exoribonucleases degrade single‐stranded RNAs (ssRNAs) from 3ʹ to 5ʹ, with PNPase and RNase PH degrading RNA by phosphorolysis, and the others by hydrolysis. The major auxiliary proteins include the DEAD‐box RNA helicases (CsdA, RhlB, DbpA, RhlE, and SrmB), the RNA pyrophosphohydrolase RppH, and the sRNA‐binding proteins (Hfq and ProQ). These proteins do not cleave or degrade RNAs directly but can alter the rate of RNA turnover catalyzed by ribonucleases.

**Table 1:** Genes encoding ribonucleases and related proteins for RNA processing or degradation in the γ‐proteobacterium *Escherichia coli*, the firmicute *Bacillus subtilis*, the unicellular cyanobacterium *Synechocystis* PCC 6803, and the filamentous cyanobacterium *Anabaena* PCC 7120.

		Genes from different bacteria				
Protein	Activity and known functions	*Escherichia coli*	*Bacillus subtilis*	*Synechocystis PCC 6803*	*Anabaena PCC 7120*	
**Endoribonucleases**						
RNase E/G	Cleaving ssRNA, participating in bulk RNA degradation	*rne*; *rng*	/	*slr1129 (rne)*	*alr4331 (rne)*	
RNase Y	Cleaving ssRNA, participating in bulk RNA degradation	/	*rny*	/	/	
RNase III	Cleaving dsRNAs, participating rRNA maturation and bulk RNA degradation	*rnc*	*rnc*	*slr1646*; *slr0346*	*alr0280*; *all4107*	
Mini‐III	Cleaving dsRNAs, mainly participating in 23S rRNA maturation	/	*mrnC*	*slr0954*	*alr1158*	
RNase HI	Cleaving the RNA strand in RNA–DNA hybrids	*rnhA*	/	*slr0080*	*alr0142*	
RNase HII	Cleaving the RNA strand in RNA–DNA hybrids	*rnhB*	*rnhB*	*slr1130*	*alr4332*	
RNase HIII	Cleaving the RNA strand in RNA–DNA hybrids	/	*rnhC*	/	/	
YbeY	Cleaving ssRNAs, mainly participating in 16S rRNA maturation	*ybeY*	*ybeY*	*slr0053*	*all0271*	
RNase P	Cleaving tRNA precursors to produce the mature 5ʹ ends	*rnpA*, *rnpB*	*rnpA*, *rnpB*	*slr1469 (rnpA)*, *rnpB*	*alr3413 (rnpA)*, *rnpB*	
RNase Z/BN	Cleaving tRNA precursors to produce the 3ʹ end for CAA addition	*rbn*	*rnz*	*slr0050*	*alr5152*	
RNase M5	Cleaving specifically at a double‐strand region in 5S rRNA precursor to produce mature 3ʹ and 5ʹ ends	/	*rnmV*	/	/	
Cas6	Cleaving specifically within the CRISPR repeats for the maturation of crRNAs	*cas6*	*cas6*	*slr7014 (cas6‐1)*; *slr7068 (cas6‐2a)*	*alr1482*; *alr1566*	
**Exoribonucleases**						
PNPase	Degrading ssRNAs from 3ʹ by phosphorolysis, polyadenylating ssRNAs	*pnp*	*pnp*	*sll1043*	*all4396*	
RNase J	Hydrolyzing ssRNAs from 5ʹ end or cleaving it at internal sites	/	*rnj1*; *rnj2*	*slr0551 (rnj)*	*all3678 (rnj)*	
RNase R/II	Hydrolyzing ssRNAs from 3ʹ end, participating in bulk RNA degradation	*rnb*; *rnr*	*rnr*	*sll1290*; *sll1910*	*all4450*; *alr1240*	
Orn	Oligoribonuclease, hydrolyzing short oligoribonucleotides into single ribonucleotides	*b4162*	/	/	/	
nanoRNase	Oligoribonuclease, hydrolyzing short oligoribonucleotides into single ribonucleotides	/	*nrnA*; *nrnB*	/	/	
RNase PH	Degrading ssRNAs from 3ʹ end by phosphorolysis, mainly producing mature tRNA 3ʹ ends	*rph*	*rph*	/	*alr0069*	
RNase D	Hydrolyzing ssRNAs from 3ʹ end, participating in tRNA maturation	*rnd*	/	*sll0320*	*all1697*; *all3791*	
RNase T	Hydrolyzing ssRNAs from 3ʹ end, mainly participating in the maturation of tRNAs, 5S RNA and 23S rRNA	*rnt*	/	/	/	
**Auxiliary proteins**						
RNA helicase	Unwinding secondary structures of double‐strand RNA segments	*rhlE*; *rhlB*; *deaD/csdA*; *dbpA*; *srmB*	*cshA*; *cshB*; *deaD/yxiN*; and *yfmL*	*slr0083 (crhR)*	*alr1223 (crhB)*; *alr4718 (crhC)*	
Hfq	Binding to sRNAs and mRNAs, facilitating the base pairing between sRNAs and their mRNA targets	*hfq*	*hfq*	*ssr3341 (hfq)*	*asl2047 (hfq)*	
ProQ	Binding mainly to mRNAs, acting as an RNA chaperone	*proQ*	/	/	/	
RppH	RNA 5ʹ pyrophosphohydrolase, converting 5ʹ triphosphate to 5ʹ monophosphate	*rppH*	*rppH*	/	/	

crRNA, CRISPR RNA; dsRNA, double‐stranded RNA; mRNA, messenger RNA; sRNA, small RNA; ssRNA, single‐stranded RNA; tRNA, transfer RNA.

**Figure 1:**
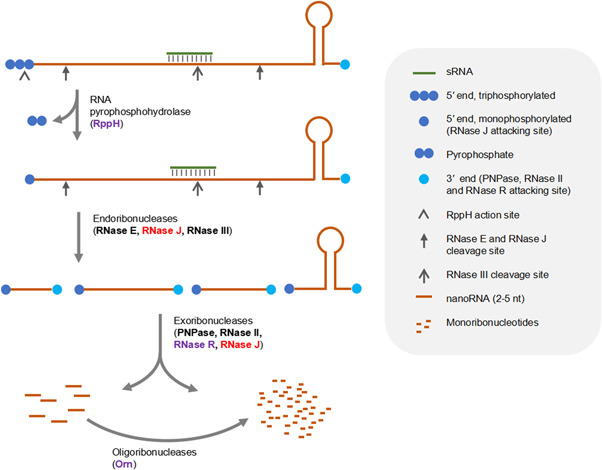
The principal pathway of messenger RNA (mRNA) degradation in bacteria. The major steps include the following: RNA phosphohydrolase converts the 5ʹ end of the primary transcripts from triphosphate to monophosphate; endoribonucleases internally cleave the transcripts into intermediate fragments; exoribonucleases degrade the intermediates into monoribonucleotides and generate the end products of 2‐ to 5‐nt oligoribonucleotides (nanoRNAs); and oligoribonucleases hydrolyze the oligoribonucleotides into monoribonucleotides, finishing the degradation process. Note that many transcripts in the triphosphorylated form can also be substrates of endoribonucleases and some transcripts may also be substrates of exoribonucleases before endoribonucleolytic cleavage. The major *Escherichia coli* and cyanobacterial enzymes involved in the degradation process are shown. The enzymes present in both *E. coli* and cyanobacteria (RNase E, RNase III, PNPase, and RNase II) are in black, those currently discovered in *E. coli* only (RppH, RNase R, and Orn) are in purple, and the one present in cyanobacteria but not in *E. coli* (RNase J) is in red. Note that RNase J acts as both an endoribonuclease and a 5ʹ−3ʹ exoribonuclease.

Tremendous insights have been gained into the bacterial RNA turnover process during the last 40 years^5^, ^6^. It is now well recognized that the RNA degradation process in bacteria takes several steps (Figure 1). In *E. coli*, the primary transcripts are first cleaved internally mainly by the endoribonuclease RNase E. Before RNase E cleavage, most primary transcripts, which are 5ʹ‐triphosphorylated, need to be converted into the RNase E preferred 5ʹ‐monophosphorylated form by the RNA pyrophosphohydrolase RppH. The fragments generated by RNase E are further trimmed from the 3ʹ end to the 5ʹ end by various exoribonucleases, such as PNPase, RNase II, and RNase R and turned into 2‐ to 5‐nt oligoribonucleotide end products (also known as nanoRNAs). These short oligoribonucleotides are eventually converted into monoribonucleotides by the 3ʹ–5ʹ oligoribonuclease Orn, completing the degradation process. No 5ʹ–3ʹ exoribonuclease has been found in *E. coli* thus far. The auxiliary proteins, Hfq, ProQ, and DEAD‐box RNA helicases, are involved in the degradation of specific RNAs through their RNA binding and unwinding activities.

As different ribonucleases participate in the degradation of one RNA, bacteria have evolved various mechanisms to coordinate their activities for efficient turnover of cellular RNAs. One of the most important mechanisms is the formation of the RNA degradosome, a protein complex whose components are mostly RNA‐degrading enzymes^7^. The core components of the RNA degradosome in *E. coli* are the endoribonuclease RNase E, the exoribonuclease PNPase, the RNA helicase RhlB, and the glycolytic enzyme enolase. RNase E acts as the scaffold to recruit other degradosomal components. It is believed that within the degradosome, the RNA fragment produced by RNase E cleavage can be quickly captured and degraded by PNPase. RhlB is able to unwind RNA duplexes to facilitate the degradation of structured substrates by RNase E and PNPase. The coupled activities of RNase E, PNPase, and RhlB ensure efficient substrate degradation and prevent the accumulation of the intermediate products. Enolase does not directly act on RNAs but can modulate the activity of the degradosome under specific conditions^8^. When the RNA degradosome is disrupted, cell growth and cellular RNA metabolism are significantly affected^9^, ^10^.

Compared to *E. coli*, *B. subtilis* presents important differences in RNA degradation systems^5^. A key difference is that *B. subtilis* does not have an ortholog of *E. coli* RNase E; instead, it has two ribonucleases that are absent in *E. coli*: RNase Y and RNase J. The endoribonuclease RNase Y is among the most important ribonucleases that control the abundance of bulk mRNAs in *B. subtilis* ^11^. It shows no detectable similarity to *E. coli* RNase E in primary sequence but has many properties remarkably similar to those of RNase E. RNase Y is able to cut the same substrates in the same way as RNase E, and the activity of both enzymes is stimulated by the presence of a 5ʹ‐monophosphate group in the substrates^12^. RNase Y is also the ribonuclease that initiates RNA cleavage *in vivo* ^13^. Last, RNase Y interacts with other proteins, such as RNA helicase and glycolytic enzyme, forming a complex that is functionally equivalent to the *E. coli* RNA degradosome^14^. RNase J is unique in that it acts as both a 5ʹ–3ʹ exoribonuclease and an endoribonuclease, and it also globally regulates RNA metabolism in *B. subtilis* ^15^, ^16^. More details regarding RNase J will be described below.

Our major understanding of bacterial RNA metabolism and ribonucleases has been obtained mainly by studies in *E. coli* and *B. subtilis*. In recent years, research interests have been extended to the RNA metabolism of other bacterial species, such as *Caulobacter*, *Helicobacter*, *Staphylococcus* and *Mycobacterium* ^17^, ^18^, ^19^, ^20^. Based on these studies, it is well recognized that different bacteria, even closely related species, may use different sets of enzymes for RNA turnover. The functional importance of the same enzyme can also vary greatly in different hosts. Thus, in addition to studying model organisms, it is very necessary to investigate the ribonucleases in species of interest, so that we can have a better understanding of RNA metabolism and gene regulation in bacteria.

### Cyanobacterial enzymes for RNA maturation and degradation

Cyanobacteria diverged from *E. coli* and *B. subtilis* over 3 billion years ago^21^. They belong to a unique bacterial phylum that comprises species with great morphological, ecological, and genetic diversity. Cyanobacteria are the only prokaryotes that carry out oxygenic photosynthesis, and many of them can also fix atmospheric nitrogen, thus contributing greatly to the carbon and nitrogen cycles in the biosphere^22^, ^23^. Therefore, cyanobacteria are excellent model organisms for studying photosynthesis and nitrogen fixation. In addition, they are being developed into efficient hosts for the renewable production of valuable products using solar energy and CO_2_ ^24^. In contrast to the importance of cyanobacteria in basic and applied studies, our understanding of ribonucleases and RNA metabolism in cyanobacteria remains very limited. Here we will summarize the current knowledge about ribonucleases and other relevant proteins in cyanobacteria and present our understanding of their roles in gene regulation at the posttranscriptional level in these organisms.

The unicellular model cyanobacterium *Synechocystis* PCC 6803 and the filamentous model cyanobacterium *Anabaena* (also called *Nostoc*) PCC 7120 were the first cyanobacterial strains to have sequenced genomes^25^, ^26^. By genomic analysis, in both strains, we identified 16 proteins homologous to the known proteins involved in RNA turnover in *E. coli* and *B. subtilis* (Table 1). Eleven of these proteins are universally present in *E. coli*, *B. subtilis*, and cyanobacteria: PNPase, RNase PH, RNase Z, RNase III, RNase H, RNase II/R, RNase P, YbeY, RNA helicases, Cas6, and Hfq. Three are present in cyanobacteria and *E. coli* but are missing in *B. subtilis*: RNase E, RNase HI, and RNase D. Mini‐III and RNase J are present only in cyanobacteria and *B. subtilis*. Some proteins (RNase E, PNPase, RNase Z, YbeY, Mini‐III, and RNase J) are encoded by single genes in each cyanobacterial genome, while others (RNase D, RNase II/R and RNase III and RNase helicase) are encoded by more than one gene. Several proteins present in *E. coli* or *B. subtilis* do not have homologs in cyanobacteria, including RNase T, Orn, RNase Y, RNase HIII, RNase M5, NrnA/NrnB, ProQ, and RppH. Taken together, cyanobacteria contain a set of ribonucleases and related proteins that are different from those used by the model bacteria *E. coli* and *B. subtilis*, implying certain unique characteristics in their RNA metabolism regulation.

To date, only a handful of proteins involved in RNA turnover has been experimentally investigated in cyanobacteria. Some of the known proteins, such as RNase P, RNase Z, RNase PH, RNase D, and Cas6, mainly participate in the maturation of stable RNAs (i.e., tRNAs, rRNAs, and crRNAs) and are functionally conserved in diverse bacteria, while the others have more important roles in mRNA turnover. In this review, we will focus on the latter.

## ENDORIBONUCLEASES

### RNase E, a key endoribonuclease in cyanobacteria

At least one of the three ribonucleases, RNase E, RNase Y, and RNase J, was found when a representative set of 1535 bacterial genomes was scanned for these genes^27^. In *E. coli,* RNase E is the most important ribonuclease for bulk RNA turnover. Its orthologs are widely distributed in Proteobacteria, Actinobacteria, Bacteroidetes, Chlamydiae, Cyanobacteria, Firmicutes, and plant chloroplasts^28^. The 1061‐aa *E. coli* RNase E can be divided into two distinct parts: the conserved N‐terminal half (NTH) and the highly divergent C‐terminal half (CTH)^29^. The crystal structure of the NTH shows that it consists of a large domain and a small domain^30^. The large domain encompasses the subdomains S1, 5ʹ‐sensor, RNase H, DNase I, and Zn‐link, with the first four conferring substrate binding and cleavage activity and the Zn‐link mediating the tetramerization of RNase E. The small domain also plays a role in tetramerization. In contrast to the compact structure of the NTH, the CTH is highly disordered. CTH is not critical for RNase E activity *in vitro*, as RNase E without this region still cleaves the substrates efficiently^31^. However, it is required for normal RNase E function *in vivo*. CTH is the scaffold of the RNA degradosome^32^, and it also contains a cell membrane‐targeting sequence that is responsible for the membrane localization of RNase E^33^.

Cyanobacterial RNase E proteins also contain a catalytic N‐terminal region and a disordered C‐terminal region^34^, ^35^. The N‐terminal region is similar to the NTH of *E. coli* RNase E, with the subdomains S1, 5ʹ‐sensor, RNase H, DNase I, and Zn‐link, but without the small domain found in *E. coli* RNase E. The C‐terminal region is also highly disordered, with no particular sequence similarity with the CTH of *E. coli* RNase E.

*E. coli* RNase E preferentially cleaves within AU‐rich regions of single‐stranded RNAs^36^, ^37^, ^38^. In the closely related *Salmonella enterica*, a uridine two nucleotides downstream of the cleavage sites was identified as an important recognition determinant for RNase E^39^. For most substrates, cleavage is efficient only when the 5ʹ end is monophosphorylated^40^, while for certain substrates, the cleavage efficiency is not affected by the 5ʹ‐end phosphorylation state^41^. The presence of both the 5ʹ‐sensing (i.e., 5ʹ‐monophosphate dependent) pathway and the direct entry (i.e., 5ʹ‐monophosphate independent) pathway of RNase E cleavage is supported by *in vivo* evidence^42^. The similarity between cyanobacterial RNase E and *E. coli* RNase E in the catalytic region suggests that they have similar catalytic activities. Indeed, cyanobacterial RNase E and its *E. coli* counterpart have the same substrate preference and cleavage pattern for all the tested substrates^34^, ^35^. Additionally, the cyanobacterial *rne* gene can complement the *rne* mutant of *E. coli*, indicating that cyanobacterial RNase E and *E. coli* RNase E have similar activity on the cellular RNA substrates^34^.

**Figure 2:**
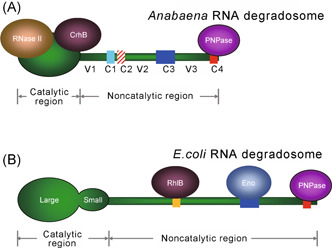
Schematic representation of the assembly of the *Anabaena* and the *Escherichia coli* RNA degradosome. (A) *Anabaena* RNA degradosome; (B) *Escherichia coli* RNA degradosome. RNase E forms tetramers; here, only one of them (in green) is shown for simplicity. The components of the degradosome are shown relative to the positions of the RNase E proteins. Four conserved subregions (C1, C2, C3 and C4) and three variable subregions (V1, V2 and V3) have been identified in the noncatalytic region of *Anabaena* RNase E^35^. Note that the *E. coli* RNase E catalytic region is composed of a large domain that is equivalent to the catalytic region of *Anabaena* RNase E and a small domain that has no counterpart in *Anabaena* RNase E.

The C‐terminal regions of cyanobacterial RNase E are much shorter and show no detectable sequence similarity to that of *E. coli* RNase E^35^. The sequences of the C‐terminal regions of all cyanobacterial RNase E proteins are also quite divergent; however, a careful alignment revealed four conserved subregions (C1, C2, C3, and C4) and three highly variable subregions (V1, V2, and V3) (Figure 2A). Three of the four conserved subregions can be detected universally in cyanobacterial homologs, but we noticed the absence of C1 in marine picocyanobacteria (*Prochlorococcus* and marine *Synechococcus*). As the *E. coli* CTH is the scaffold of the RNA degradosome, the C‐terminal region of cyanobacterial RNase E could have a similar role *in vivo*. Indeed, cyanobacterial RNase E was found to interact with PNPase via the Arg‐rich C4 subregion, forming a complex resembling the *E. coli* RNA degradosome^35^. The cyanobacterial degradosome is distinct from the *E. coli* degradosome in composition and the way of assembly (see more details below). The functions of the other subregions are currently unknown but we noticed that C2 is almost as Arg‐rich as C4. Moreover, for two residues in the variable subregion V2 of the *Synechocystis* PCC 6803 enzyme, K494, and K512, cross‐linking to an RNA substrate was observed^43^, pointing at possible functions in the positioning of the substrate.

*E. coli* RNase E is localized to the cell membrane via a 15‐residue membrane‐targeting motif forming an amphipathic α‐helix^33^. Disruption of its membrane localization by removing the targeting motif resulted in slow growth, indicating abnormal cellular RNA metabolism^33^. In the sequences of cyanobacterial RNase E, no membrane‐targeting motif can be detected. It has been shown that the RNase E of *Anabaena* PCC 7120 is in fact a cytoplasmic protein^44^. In the proteobacterium *Caulobacter crescentus*, which is evolutionally far from cyanobacteria but close to *E. coli*, RNase E also displays cytoplasmic localization^45^. Apparently, RNase E has evolved specific subcellular localization in different cellular contexts. Another potentially highly interesting interaction between RNase E and RNA has recently been reported, showing that the enzyme could be modified by the addition of a short RNA chain (called RNAylation)^46^. This observation requires further work, but if confirmed, might have far‐reaching implications.

The physiological function of RNase E has been experimentally investigated in a few cyanobacterial species. The *rne* gene was found to be indispensable in *Synechococcus* PCC 7002 and *Synechocystis* PCC 6803^47^, ^48^, and our attempt to inactivate the *rne* gene in *Anabaena* was also unsuccessful. Thus, the *rne* gene is essential in cyanobacteria. In *Anabaena* PCC 7120, overexpressing either the full‐length or the catalytic region of RNase E led to severe growth inhibition and changes in cell morphology^49^. In an *rne* partial mutant of *Synechocystis* PCC 6803, more than 2000 genes showed altered expression^48^. These studies indicate a global role of RNase E in cyanobacteria.

An experimental approach to characterize the targetome of an essential RNase requires the construction of a strain expressing a temperature‐sensitive enzyme. Then, transcriptomes are analyzed by RNA‐seq before and after transfer of the mutant strain to the nonpermissive temperature and compared with each other and with a wild‐type control. This approach is called “transient inactivation of an essential ribonuclease followed by RNA‐seq” (TIER‐seq)^39^ and has been applied recently to the transcriptome‐wide identification of RNase‐E‐dependent cleavage sites in *Synechocystis* PCC 6803^50^. One challenge in engineering a temperature‐sensitive RNase E in this cyanobacterium was that the introduction of the amino acid substitution G63S or I65F, which corresponds to G66S or L68F that renders the *E. coli* enzyme temperature‐sensitive^51^, was not sufficient. Instead, one of three additional mutations (V94A, V297A, or G281E) was needed to obtain viable mutant strains. Using one of such mutant strains for TIER‐seq analysis, 1472 RNase‐E‐dependent cleavage sites were mapped transcriptome‐wide in *Synechocystis* PCC 6803^50^. Careful analysis of these sites revealed enrichment for adenine residues at positions ‐4 and ‐3 upstream and uridine residues immediately downstream of the cleavage site, especially at position +2. These conserved sites could form an AU clamp, which is a possible signal for positioning the actual cleavage site^50^. Moreover, the uridine preferred at the +2 position in an AU‐rich RNA sequence stretch matches the target preference identified for the RNase E of *Salmonella enterica* ^39^. Given the ability of RNase E to cleave potentially thousands of sites, it is important to understand how the specificity and target recognition of RNase E is modulated. Specific proteins mediating target selection were identified in *E. coli*, such as Hfq (see below) or the RNase adaptor protein RapZ, which binds to the sRNA *GlmZ* ^52^, ^53^, ^54^. RapZ senses the cell envelope precursor glucosamine‐6‐phosphate^55^ and has been found to boost RNase E activity in an intriguing way, through interaction with its catalytic domain^56^. Potential homologs of RapZ exist in several cyanobacteria (e.g., NJM11451, REJ55829, and WP_071838815, all with >60% similar residues), but none of these proteins has been functionally characterized thus far.

Interestingly, in addition to its role in bulk RNA metabolism, *Synechocystis* RNase E also participates in the maturation of crRNAs^43^. In most CRISPR‐Cas systems, specialized endoribonucleases, often belonging to the Cas6 family, mediate the maturation of crRNAs. In the case of the type III‐Bv CRISPR system in *Synechocystis* PCC 6803, RNase E was found to recognize the stem‐loop region of the precursor crRNA, followed by cleavage within the downstream single‐stranded region to produce the mature crRNA^43^. This is one of the few known cases where crRNA is not matured by a Cas protein.

**Figure 3:**
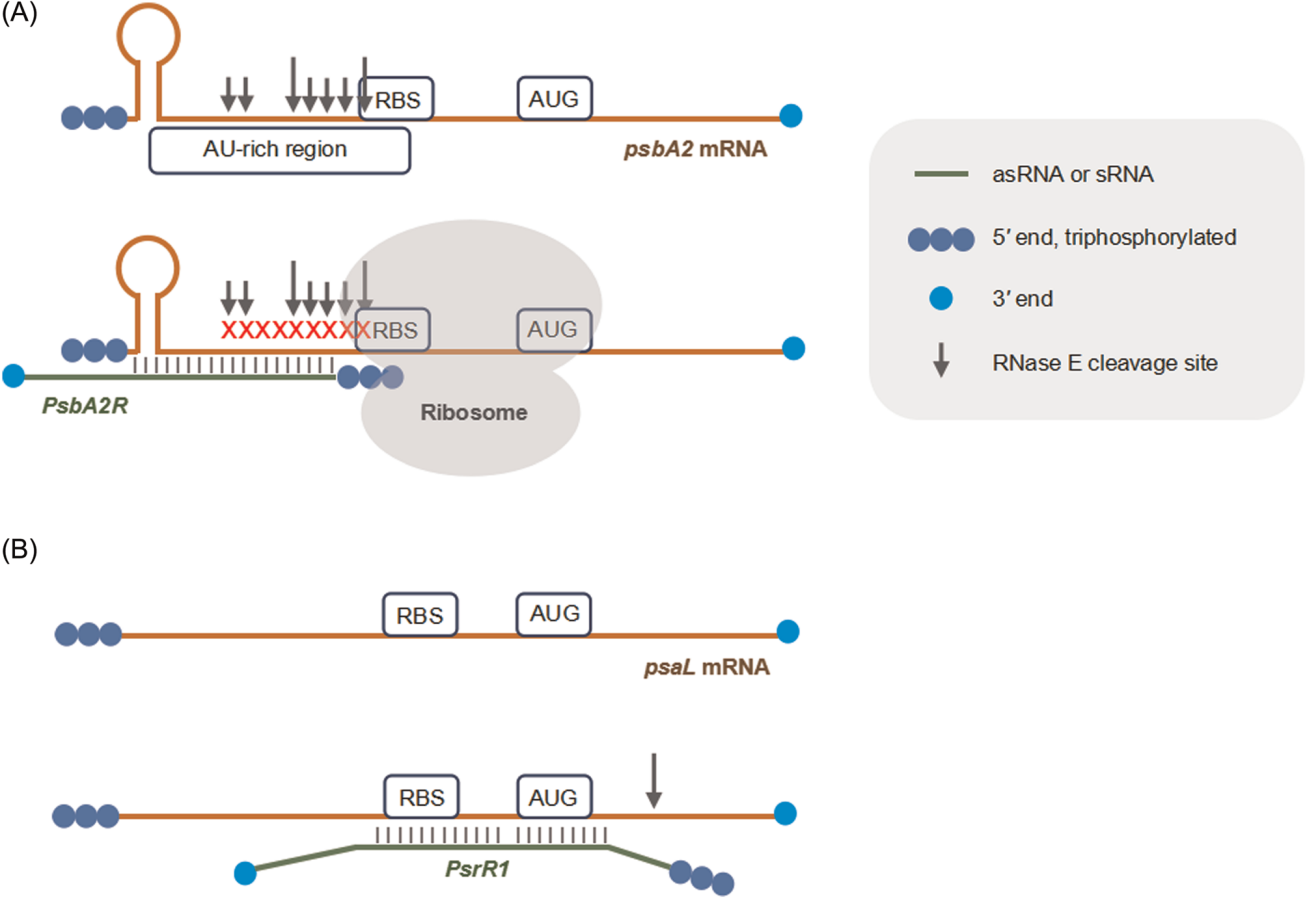
RNase E cleavage of messenger RNAs (mRNAs) encoding photosynthetic proteins in *Synechocystis* PCC 6803. (A) Upper panel: The *psbA2* mRNA has a 49‐nt 5ʹ UTR that, in the dark, is sensitive to RNase E cleavage due to multiple RNase E cleavage sites within an AU‐rich sequence that extends into the likely ribosome binding site (RBS)^43^, ^50^, ^57^. Hence, rapidly interacting ribosomes under conditions such as high light, when this mRNA is strongly translated, can protect against degradation (lower panel). In addition, the antisense RNA (asRNA) *PsbA2R* is coregulated with the mRNA from a transcriptional start site leading to an overlap with the first 19 nucleotides of the *psbA2* mRNA, hence protecting these sites against RNase E cleavage^58^, as indicated by the red crosses. A short stem‐loop near the mRNA 5ʹ end might be relevant for recognition. Note that *in vivo* additional ribonucleases are likely involved^50^. Note that the gene *psbA3*, which is almost identical to *psbA2*, is regulated in the same way^57^, ^58^. (B) Upper panel: The *psaL* gene is preceded by a 55‐nt 5ʹ UTR that is not targeted by RNase E under most growth conditions. However, under high light, transcription of the gene for the sRNA *PsrR1*, which is located elsewhere in the genome, is stimulated. Except for two mismatches, *PsrR1* interacts over 22 consecutive nucleotides with the *psaL* mRNA (lower panel). This interaction overlaps the 3ʹ end of the 5ʹ UTR, including the ribosome binding site, the start codon, and one additional nucleotide of the second codon. This interaction leads to conditional recruitment of RNase E, which then cleaves a single‐bond seven nucleotides into the coding sequence, effectively decapitating the *psaL* mRNA^59^.

Transcripts encoding photosynthesis proteins are a major target of RNase E in *Synechocystis* PCC 6803^50^. The relation between RNase E and some of these transcripts has been studied in more detail, such as for *psaL*, which encodes the photosystem I reaction center protein XI, or the *psbA2* and *psbA3* genes that encode the photosystem II reaction center protein D1 (Figure 3). The almost identical *psbA2* and *psbA3* transcripts are not stable in the dark due to the presence of an AU‐box within their 5ʹ UTRs. As RNase E could efficiently cleave within the AU‐box *in vitro*, it was thus suggested that it is responsible for the rapid degradation of those AU‐box containing transcripts in the dark^57^. This discovery raised the question of how the activity of RNase E on these transcripts would be prevented under other conditions. A potential answer lies in the fact that the AU‐box overlaps with the region for the initiation of translation. Thus, interacting ribosomes on these mRNAs that are strongly translated in the light would protect against degradation. This model was further refined by the discovery of weak promoters in *Synechocystis* PCC 6803 on the reverse strand that give rise to cis‐antisense RNAs called *PsbA2R* and *PsbA3R*. Since these antisense RNAs are coregulated with the *psbA*2/3 mRNAs, they protect the RNase E target sites not covered by ribosomal subunits^58^. An interplay between RNase E and an mRNA modulated by a noncoding RNA was also discovered for *psaL*. This gene encodes the photosystem I reaction center protein XI, which is involved in the trimerization of photosystem I. Under high light conditions, the monomeric status is the preferred configuration; hence, *psaL* expression needs to be reduced. Under this condition, transcription of the sRNA *PsrR1* is stimulated. *PsrR1* interacts with a target region in the *psaL* mRNA; upon binding, RNase E is recruited to this site, which then cleaves at a site seven nucleotides into the coding sequence^59^.

An intriguing mechanism was discovered for the upregulation of RNase E expression in marine picocyanobacteria. During lytic infection of *Prochlorococcus* MED4 with the T7‐like cyanophage P‐SSP7, *rne*, the gene encoding RNase E was one of very few upregulated host genes. This result was interpreted as a mechanism by which the phage could benefit from the stimulated degradation of host transcripts for use as substrates for phage deoxynucleotide synthesis^60^. To achieve upregulation, an alternative transcription start site was identified that was only activated during infection and from which an mRNA was derived that lacked the 5ʹ UTR, thereby bypassing the normally tight control of RNase E expression^61^.

### Most cyanobacteria having more than one RNase III gene

RNase III family proteins are endoribonucleases that specifically cleave dsRNAs. They are widely distributed in Bacteria, Archaea, and Eukarya. Based on their sequence features, bacterial RNase III family proteins can be grouped into two categories. The first category includes the canonical homodimeric RNase III enzymes, with each subunit containing one RNase III domain and a dsRNA binding domain (dsRBD). The second category corresponds to the Mini‐III enzymes, which also act as a dimer, but each subunit consists of a single RNase III domain only^62^. Here, we refer to the enzymes in these two groups as RNase III and Mini‐III, respectively.

*E. coli* and *B. subtilis* each have only one gene encoding RNase III. In contrast, different cyanobacterial species usually have 1 to 3 RNase III‐encoding genes. For instance, two RNase III family proteins (A0061 and A2542) are present in *Synechococcus* PCC 7002, and both can cleave many substrates of *E. coli* RNase III, although they do not always cleave at the same sites as *E. coli* RNase III^47^. A0061 was shown to be involved in the maturation of tRNAs and rRNAs^63^. Note that *E. coli* RNase III also plays an important role in rRNA maturation^64^, ^65^. Thus, maturing stable RNAs may be a common function of bacterial RNase III proteins. Disruption of the gene encoding A2542, the other RNase III family protein in *Synechococcus* PCC 7002, did not affect rRNA maturation but greatly increased the copy number of the endogenous plasmid pAQ3^63^. The mechanism of plasmid copy number control by A2542 remains unclear. *Anabaena* PCC 7120 also has two RNase III family proteins. One of them (Alr0280) could cleave an artificial dsRNA, while the other (All4107) could not^66^. We noticed that the start codon of *all4107* was misidentified in the original annotation, and the recombinant All4107 prepared by Gao et al.^66^, which is based on this incorrect annotation, lacks the first 40 residues. This is probably the reason why All4107 was inactive in their studies.

Although RNase III family proteins participate in the maturation and degradation of many cellular RNAs, they are not required for the viability of *E. coli* and many other bacteria^67^, ^68^, ^69^. One noticeable exception is *B. subtilis* RNase III, which is essential for cell growth^70^. It is now clear that the essentiality of *B. subtilis* RNase III results from its activity toward the toxin genes borne by two prophages and not from its influence on the metabolism of other cellular RNAs^71^, ^72^. In cyanobacteria, RNase III proteins are likely also dispensable. Loss of each of the two RNase III proteins, or of both, in *Synechococcus* PCC 7002 did not significantly affect cell growth, although it led to altered expression of hundreds of genes^47^, ^63^. In *Anabaena* PCC 7120, at least one of the RNase III‐encoding genes, *alr0280*, could be inactivated; however, the phenotype of the mutant was not mentioned^66^. A significant share of the bacterial transcriptome consists of overlapping antisense transcripts (for review, see reference^73^). In cyanobacteria, some antisense transcripts were demonstrated to control the level of the respective overlapping mRNA in a codegradation mechanism, such as for the *isiA* gene in *Synechocystis* PCC 6803 under iron starvation^74^. RNase III was found to be the enzyme responsible for such codegradation in bacteria^75^. In cyanobacteria, this was clearly demonstrated for an antisense RNA called *as_glpX* that is transcribed in *Anabaena* PCC 7120 within the gene *glpX* encoding sedoheptulose‐1,7‐bisphosphatase/fructose‐1,6‐bisphosphatase (SBPase). The *as_glpX* transcript is specifically transcribed in heterocysts and the RNase III Alr0280 was demonstrated to mediate the codegradation of *as_glpX* and its cognate mRNA^76^. The outcome of this codegradation is a reduced amount of SBPase contributing to the shutdown of the Calvin cycle specifically in those cells that are undergoing differentiation into heterocysts. RNase III‐encoding genes are found in all sequenced cyanobacterial species, and such a wide distribution suggests that they may be required for the acclimation of cyanobacteria to fluctuating environments.

The activity of *E. coli* RNase III was shown to be regulated by the *O*‐acetyl‐ADP‐ribose deacetylase YmdB^77^, ^78^. YmdB homologs are present in a few cyanobacteria, including *Synechocystis* PCC 6803, where it is fused as a C‐terminal domain to a YfbK‐domain‐containing protein, encoded by *slr7060* on plasmid pSYSA. It would be interesting to test whether RNase III proteins could be regulated by YmdB homologs in these cyanobacterial species.

Although absent in *E. coli*, Mini‐III family proteins are also widely distributed. Mini‐III was originally identified and characterized in *B. subtilis* ^79^. It mainly participates in the maturation of the 23 S rRNA precursor^79^, ^80^. Genes encoding a Mini‐III family protein are present in every sequenced cyanobacterial species. Mini‐III in *Synechococcus* PCC 7002 was found to mature 23S rRNA^63^. Likewise, in the plant chloroplast, Mini‐III is involved in the maturation of 23S rRNA, 16S rRNA, and 4.5S rRNA^81^. Thus, maturation of rRNAs (particularly 23S rRNA) is likely a function common to Mini‐III family proteins. *B. subtilis* Mini‐III seems to have much more stringent substrate specificity than canonical RNase III, as it has been shown to cut dsRNA preferentially at the ACC^U site^82^. The substrate specificity of Mini‐III proteins from cyanobacteria and other bacteria has not yet been determined.

### YbeY, one of the most conserved bacterial endoribonucleases

The endoribonuclease YbeY, which cleaves both single and double‐stranded RNA substrates, is one of the most conserved proteins in bacteria^83^. In *E. coli*, *Sinorhizobium meliloti* and many other bacteria, YbeY participates in the maturation of ribosomal RNAs (particularly 16S rRNA)^84^, ^85^, ^86^, ^87^. In addition, YbeY was also shown to be involved in sRNA‐mediated mRNA degradation, probably by directly cleaving the sRNA‐mRNA duplex^88^, ^89^. YbeY‐encoding gene is present in every sequenced cyanobacterial genome, and the high conservation of YbeY in bacteria suggests that this protein may also function in rRNA mutation and posttranscriptional gene regulation in cyanobacteria.

### RNase H family proteins

RNase H family proteins are the primary enzymes responsible for recognizing and cleaving RNA–DNA hybrids. Such hybrids are prevalent in cells and can compromise genome integrity if not eliminated. RNA–DNA hybrids exist in several forms *in vivo*, including the R‐loop, which occurs during transcription when the nascent RNA strand bases pair with its template DNA; covalently incorporated single ribonucleotides or short polymers of RNA due to DNA replication error; and the oligoribonucleotides used to prime the synthesis of Okazaki fragments^90^, ^91^, ^92^, ^93^. RNase H family proteins, which specifically remove these RNA–DNA hybrids, are widely present in bacteria and can be classified into two groups based on sequence similarity: class one, which is represented by RNase HI, and class two, which consists of RNase HII and RNase HIII^94^. RNase HI cleaves within RNA–DNA hybrids and such substrates should have four or more consecutive ribonucleoside monophosphates (rNMPs). RNase HII has been shown to cleave 5ʹ to single embedded rNMPs or 3ʹ to the first rNMP at an RNA‐DNA junction when multiple rNMPs are present^95^, ^96^. It should be noted that the genes encoding RNase HII in many cyanobacteria occur in a conserved dicistron together with the gene encoding RNase E.

Although RNase HIII is more closely related to RNase HII in sequence, its activity is very similar to that of RNase HI, cleaving RNA–DNA hybrids with three or more consecutive rNMPs^97^. As RNase HI and RNase HIII have similar activities, most bacteria have either RNases HI and HII, or HII and HIII^98^. For example, *E. coli* has RNase HI (encoded by *rnhA*) and RNases HII (encoded by *rnhB*), and *B. subtilis* has RNase HII and RNase HIII (encoded by *rnhC*). In *E. coli*, RNase HI mainly removes R‐loops by cleaving the RNA strand in the RNA–DNA hybrids, generating RNA primers that could either be used by DNA polymerase I (Pol I) for the synthesis of Okazaki fragments or be degraded by Pol I^99^, ^100^. RNase HII is involved in removing the misincorporated rNMPs in genomic DNA^92^, ^93^, ^101^. While inactivation of *rnhB* did not result in an obvious growth defect, inactivation of *rnhA* led to slow growth *in E. coli* ^93^, ^102^. Furthermore, the inactivation of both genes resulted in much poorer growth and impaired chromosome replication and segregation^103^, suggesting the critical roles of these two RNase H proteins *in vivo*. An RNase HI and an RNase HII are encoded by each sequenced cyanobacterial genome, and they both show high sequence similarities to their *E. coli* counterparts. It is likely that they also play important roles in eliminating R‐loops and misincorporated rNMPs from genomic DNA.

## EXORIBONUCLEASES

### RNase J and its enigmatic role in cyanobacteria

RNase J was originally identified in *B. subtilis* ^104^ in which two RNase J family proteins exist, RNase J1 (encoded by *rnjA*) and RNase J2 (encoded by *rnjB*), which can form heterodimers *in vivo* ^105^. RNase J1 acts as both an endoribonuclease and an exoribonuclease; however, structural analyses suggest that it mainly acts as an exoribonuclease^106^, ^107^. This enzyme is the first 5ʹ–3ʹ exoribonuclease identified in bacteria^15^. RNase J2 has much lower activity than RNase J1, although the two proteins show high sequence similarity^105^. Both *rnjA* and *rnjB* can be deleted in *B. subtilis* but the consequences are quite different. While the deletion of *rnjB* did not affect growth, that of *rnjA* led to severe growth defects, indicating that RNase J1 is much more important than RNase J2 *in vivo* ^16^. A recent study showed that RNase J could resolve the stalled RNA polymerase (RNAP) complex from DNA by degrading the nascent RNA in the complex and finally colliding the RNAP off DNA, thereby playing an important role in preventing transcription–replication collisions^108^.

RNase J family proteins are present in many archaeal and bacterial species^104^. However, the number of RNase J homologs and their importance in different organisms may vary significantly. For instance, two RNase J homologs are present in the pathogen *Staphylococcus aureus*, and although they also seem to function as a complex *in vivo,* deletion of either of them could greatly affect growth^109^, ^110^. The only RNase J homolog in the gastric pathogen *Helicobacter pylori* is essential for viability, and it was shown to associate with the DEAD‐box RNA helicase RhpA, forming a minimal RNA degradosome^111^. A single RNase J‐encoding gene, *rnj*, is present in every sequenced cyanobacterial genome. Gene expression of *rnj* in *Synechocystis* PCC 6803 was found to respond to a variety of environmental stresses such as iron starvation^112^, or to the absence of the RNA helicase CrhR^113^. The gene (ID *slr0551*, cf. Table 1) has a very long 5ʹ UTR of 455 nt^114^, which is also detectable as a separate transcript in the cell and may function as an sRNA (called NC‐117^115^). To date, limited investigation of the function of RNase J has been carried out. RNase J from *Synechocystis* PCC 6803 has catalytic properties very similar to those of *B. subtilis* RNase J1: as an exoribonuclease that degrades RNAs whose 5ʹ end is not triphosphorylated, and as an endoribonuclease that cuts substrates at sites where *B. subtilis* RNase J1 does^48^. Attempts to inactivate *rnj* in several cyanobacterial species have been unsuccessful, implying that RNase J is essential in cyanobacteria^47^, ^48^, ^116^. In a partially segregated *rnj* mutant of *Synechocystis* PCC 6803, the maturation of the CRISPR3 crRNA was affected, but unlike RNase E, there was no evidence provided that RNase J could directly act on the precursor crRNA^48^. In this partial mutant, only 180 genes, most of which are located on endogenous plasmids, had altered expression^48^. The small number of genes affected may be due to the insufficient depletion of RNase J in this strain. Given that RNase J is essential in cyanobacteria, it is expected to have a global role in gene regulation.

Existing knowledge of RNase J in cyanobacteria suggests that it is a conserved and essential enzyme, but its true functionality has remained unknown thus far. In contrast, the functions of RNase J in plant chloroplasts are understood much better. Plant RNase J is nucleus‐encoded but localized in chloroplasts. Its catalytic properties are similar to those of *B. subtilis* RNase J1^117^. An *Arabidopsis* mutant without RNase J could not form normal chloroplasts, resulting in aberrant embryo development^118^, ^119^. Massive accumulation of antisense RNAs was observed when RNase J was depleted from the chloroplast^120^, and it is likely that RNase J plays a key role in 5ʹ end maturation of chloroplast mRNAs^121^. Because of the cyanobacterial origin of chloroplasts, knowledge of chloroplast RNase J could provide valuable insights into the functions of RNase J in cyanobacteria.

### RNase II/R

Two RNB family 3ʹ−5ʹ exoribonucleases are present in *E. coli*: RNase II (encoded by *rnb*) and RNase R (encoded by *rnr*). RNase II only degrades the 3ʹ‐single‐stranded region of substrates^122^, while RNase R, which has RNA helicase activity, can efficiently degrade structured RNAs that have a single‐stranded tail^123^, ^124^. The two enzymes have different cellular substrates in *E. coli*. RNase II is mainly involved in the degradation of mRNAs^125^, although it was also shown to protect *rpsO* mRNA from attack by other exoribonucleases by removing its poly(A) tail appended by poly(A) polymerase^126^. In contrast, RNase R plays an important role in the degradation of rRNAs and mRNAs with complicated structures^123^, ^127^. *B. subtilis* has only one RNB family protein, RNase R, which, similar to *E. coli* RNase R, is capable of degrading structured RNAs^128^.

Each sequenced cyanobacterial genome contains two or three RNB family exoribonucleases. In *Synechocystis* PCC 6803, one of the RNB family proteins, Sll1290, has the same substrate specificity as that of *E. coli* RNase II and only degrades ssRNAs while producing 4‐nt end products^129^. Whereas *E. coli* RNase II prefers polyadenylated substrates, Sll1290 does not have such a preference, in line with the fact that cyanobacterial mRNAs do not have a homogenous poly(A) tail^130^. Another cyanobacterial RNB family protein, Alr1240, from *Anabaena* PCC 7120 also has properties similar to those of *E. coli* RNase II, as it can only degrade unstructured substrates^44^. It is still unclear whether RNase R‐like RNB family exoribonucleases exist in cyanobacteria.

RNB family proteins are dispensable in *E. coli* and *B. subtilis* as their activities can be replaced by other exoribonucleases^125^, ^128^, ^131^. In contrast, the essentiality of RNB family proteins in cyanobacteria seems to be species dependent. In *Synechocystis* PCC 6803, *sll1290* is indispensable^129^, while the other RNB protein‐encoding gene *sll1910* could be inactivated, and the resulting strain showed resistance to the herbicide acetazolamide^132^. Similarly, only one of the two RNB‐family genes in *Synechococcus* PCC 7002 could be inactivated and the mutant strain grew only slightly slower than wild type^47^. In contrast, each of the RNB‐family protein‐encoding genes in *Synechococcus* PCC 7942 could be inactivated by transposon mutation^116^.

Recently, the *Anabaena* PCC 7120 RNB family protein Alr1240 was shown to form a complex with RNase E, and these two proteins colocalized in the cytoplasm^44^. Alr1240 enhanced substrate cleavage activities by RNase E *in vitro* ^44^. These results suggest that Alr1240 and RNase E act together *in vivo* and that Alr1240 may have a regulatory role in the activity of RNase E.

### PNPase

PNPase, encoded by the *pnp* gene, is an exoribonuclease that catalyzes the phosphorolysis of RNAs from 3ʹ‐ends, and it can also catalyze the reverse reaction that adds heteropolymeric tails to RNA 3ʹ ends^133^. This protein is widely distributed in bacteria, chloroplasts and mitochondria, but is missing in Archaea^134^. PNPase processively degrades RNAs with 10‐ to 12‐nt long single‐stranded 3ʹ ends. Similar to RNase II, the action of PNPase is easily stopped by stable secondary structures in RNAs^135^, ^136^. Nevertheless, PNPase can cooperate with the RNA helicase RhlB, either by directly forming the PNPase‐RhlB complex or coexisting in the RNA degradosome, to degrade structured RNAs^137^, ^138^.

Together with RNase II and RNase R, PNPase plays a global role in exoribonucleic degradation of cellular RNAs in *E. coli*, and its depletion could influence the expression of more than 1000 genes^139^, ^140^, ^141^, ^142^. Although dispensable for growth under standard conditions, it is essential for growth at low temperatures in *E. coli* ^143^, ^144^. Such a function could be attributed to the role of PNPase in rRNA maturation and ribosome biogenesis, as defects in ribosome biogenesis are often associated with cold sensitivity^145^, ^146^, ^147^, ^148^, ^149^. PNPase also selectively degrades the mRNAs of cold shock proteins after the low‐temperature acclimation phase to allow cells to resume growth^150^. In many other bacteria, including *B. subtilis*, PNPase was also shown to be necessary for survival at low temperatures^144^, ^151^, ^152^, suggesting that it has a common function in these organisms.

Cyanobacterial PNPases have not yet been biochemically investigated. However, they are close to *E. coli* PNPase in sequence (e.g., *Anabaena* PNPase shares 48% identities and 64% similarities with its counterpart in *E. coli*), and such a high similarity suggests conserved catalytic properties. Cyanobacterial PNPases also interact with RNase E *in vivo* within the RNA degradosome^33^. Inactivation of the *pnp* gene in *Synechocystis* PCC 6803 and *Synechococcus* PCC 7002 was not successful^47^, ^130^, suggesting that PNPase has much more important physiological roles in cyanobacteria than in *E. coli*. Considering that cyanobacteria do not possess the homologs of poly(A) polymerase, which is the major enzyme for RNA polyadenylation in *E. coli* ^153^, PNPase is likely the only enzyme that adds heterogeneous poly(A)‐rich tails to RNA molecules important for efficient degradation^130^.

## AUXILIARY PROTEINS

### DEAD box RNA helicases

DEAD‐box RNA helicases are the major RNA helicases in bacteria. They facilitate the degradation of structured RNAs by unwinding double‐stranded regions, particularly under low‐temperature conditions. In *E. coli*, the DEAD‐box helicases RhlB and CsdA are found in the RNA degradosome^154^, ^155^. In addition to their roles in RNA degradation, DEAD‐box helicases also participate in other biological processes, such as ribosome biogenesis and the regulation of translational initiation^156^.

Many bacteria have multiple DEAD‐box helicases. For instance, *E. coli* and *B. subtilis* have five and four DEAD‐box proteins, respectively. Cyanobacteria usually have 1 to 3 genes encoding DEAD‐box helicases, except that *Synechococcus* PCC 7942 and a few other species have no such genes^157^. According to their sequence similarity, cyanobacterial DEAD‐box helicases have been classified into three groups: CsdA‐like, RhlE‐like, and CrhR‐like^157^.

The only DEAD‐box RNA helicase in *Synechocystis* PCC 6803, CrhR, has been extensively studied in recent years. CrhR is a bidirectional, ATP‐stimulated RNA helicase that unwinds the RNA duplex from either 5ʹ–3ʹ or 3ʹ–5ʹ; additionally, it also catalyzes the formation of RNA duplexes by annealing two complementary RNA strands^158^. By combining unwinding and annealing activities, CrhR is able to catalyze RNA strand exchange^158^. Temperature is the environmental factor that significantly influences the expression of *crhR*. When transferred from standard growth temperature (30°C or 34°C) to a low temperature (24°C or 20°C), both the *crhR* transcript and the CrhR protein accumulate greatly in cells^159^, ^160^. At 30°C, CrhR could be degraded rapidly by an unknown protease *in vivo* ^161^.

At 30°C or 34°C, the *crhR* mutant of *Synechocystis* PCC 6803 did not show altered growth, and it only had a small number of genes with altered expression^113^, ^159^, ^162^, consistent with the low level of expression of *crhR* under these conditions. However, the mutant showed a retarded growth at 24°C and lost viability at 20°C^159^, ^163^, ^164^. One conclusion from these studies was that under low temperature, CrhR is required to maintain efficient photosynthesis. Consistent with this conclusion, RNA coimmunoprecipitation experiments with extracts from *Synechocystis* PCC 6803 strains expressing FLAG‐tagged CrhR yielded a striking bias toward photosynthesis‐associated and redox‐controlled transcripts^165^. In addition, the transcription and mRNA stability of *crhR* were also shown to be correlated with the redox state of the electron transport chain between *Q*_A_ in photosystem II and *Q*_O_ in cyt *b6f* ^166^. While the plastoquinone pool was in the reduced state in wild type, it was in the oxidized state in the *crhR* mutant. Additionally, the amount of PSI trimers, the transcript levels of *psaA* and *psaB*, the PSII activity, and the carbon fixation rate all decreased in the mutant^163^, ^164^. Analysis of intracellular structures by scanning electron microscopy (SEM) indicated that the architecture of ribosomes, carboxysomes, and thylakoid membranes were all highly disorganized^164^. How CrhR depletion leads to these physiological and structural changes in the cells remains unknown. A further interaction between CrhR and RNA metabolism was found when RNase E was identified as the endoribonuclease involved in the autoregulation of *crhR* expression and operon discoordination in the *rimO‐crhR* operon^165^, ^167^.

*Anabaena* PCC 7120 has two RNA helicases: CrhB (CrhR‐like) and CrhC (RhlE‐like). The enzymatic properties of CrhC have been characterized *in vitro*. Unlike the bidirectional CrhR, ChrC only unwinds the RNA duplex from 5ʹ to 3ʹ; it also lacks annealing activity^168^. Nevertheless, the expression of *crhC* is also cold inducible. The *crhC* transcript was highly unstable at higher temperatures and it was detectable only when the cells were grown at temperatures lower than 25°C^169^, ^170^. CrhC was shown to be localized to the cytoplasmic membrane, although it had no discernable membrane‐targeting motif^171^. CrhC may function with other proteins *in vivo*, as a coimmunoprecipitation assay showed that CrhC coprecipitated with several other proteins, and one of them was further shown to interact with CrhC directly^168^. However, the identities of the coprecipitated proteins were not further determined.

Another *Anabaena* helicase, CrhB, shows constant expression levels in a wide range of temperatures (at least from 20°C to 44°C), and under various stress conditions, including darkness, nitrogen starvation, and salt stress^169^. The constitutively expressed CrhB and the cold‐inducible ChrC may have distinct biological functions. A recent study showed that CrhB could interact with RNase E, and thus it may be one component of the cyanobacterial RNA degradosome^172^. The exact roles of CrhB and CrhC in RNA turnover in *Anabaena* PCC 7120 remain to be discovered.

### Hfq, not binding RNA in cyanobacteria

Hfq is one of the most important RNA chaperones in bacteria. Although Hfq does not degrade RNA, it can bind to small RNAs (sRNAs) and promote annealing between sRNAs and their target mRNAs, thereby regulating the stability and translation of these mRNAs^173^, ^174^. In fact, Hfq is now recognized as a global regulator that controls cell fitness under various conditions^175^, ^176^. Another RNA chaperone of similar functional relevance as Hfq in the Enterobacteriaceae, which was more recently recognized, is ProQ^177^. For a review on the differences and similarities between Hfq and ProQ and the comparison to other enterobacterial RNA‐binding proteins, see reference^178^.

Hfq homologs are present in most cyanobacteria, except in obligate symbionts and some *Prochlorococcus* strains^179^. Within the *Prochlorococcus* group, an interesting discrepancy was observed. While strains such as MIT9313, which represent a more deeply rooted subclade, possess an *hfq* gene, other strains with a more streamlined genome, such as MED4, lack an *hfq* homolog, although the genomic region flanking *hfq* is otherwise conserved^180^. Although cyanobacterial and *E. coli* Hfq proteins share a low similarity in primary sequence, they are strikingly similar in tertiary structure^181^. However, genetic and biochemical assays indicated that cyanobacterial Hfq proteins are unlikely to be related to RNA metabolism. First, the cyanobacterial Hfq lacks the key residues involved in RNA binding found in the Hfq proteins of other bacteria, and it has very low affinity to RNA; second, the cyanobacterial *hfq* gene could not complement the *E. coli hfq* mutant; and third, inactivation of cyanobacterial *hfq* did not influence cell growth^181^. In fact, it is now clear that Hfq in cyanobacteria functions as a regulator of cell motility instead of being an RNA chaperone. Inactivation of *hfq* in *Synechocystis* PCC 6803 led to the loss of cell motility and pili formation^182^. Hfq, together with PilB and EbsA, was recently shown to form a tripartite complex that regulates the biogenesis of type IV pili^183^.

Although cyanobacterial Hfq does not function as the sRNA‐binding RNA chaperone, sRNAs are prevalent in cyanobacteria, and several of them were shown to regulate gene expression at the posttranscriptional level^59^, ^114^, ^184^, ^185^, ^186^, ^187^, ^188^. The efficient regulation mediated by these sRNAs suggests that non‐Hfq‐type RNA chaperones exist in cyanobacteria.

### Novel RNA‐binding protein candidates in cyanobacteria

**Figure 4:**
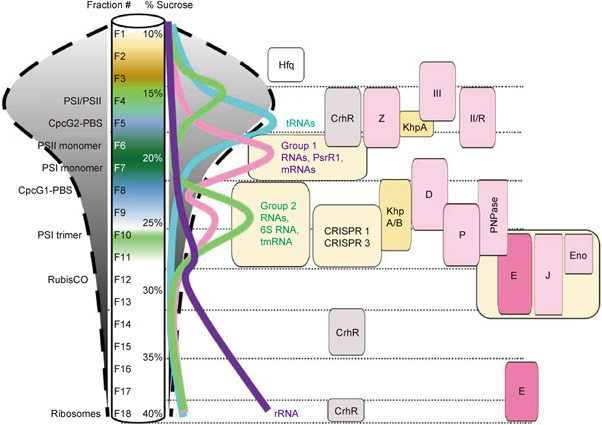
Grad‐seq analysis aids the analysis of ribonucleases, auxiliary proteins, and ribonucleoprotein complexes. A typical sedimentation profile obtained in the analysis of the cyanobacterium *Synechocystis* PCC 6803 is shown^190^. The positions of several major macromolecular complexes as determined by mass spectrometry are given to the left, the respective fraction numbers and sucrose percentages are indicated along the gradient. The different colors result from the native pigmentation of protein–pigment complexes involved in photosynthesis. The distribution of distinct groups of RNAs is sketched by the colored lines to the right of the gradient. The positions of abundant RNA–protein complexes, such as two of the three CRISPR complexes^193^ and noncoding RNA–ribonucleoprotein complexes containing 6S RNA or transfer‐messenger RNA (tmRNA) are shown. Note that characterized regulatory small RNAs such as *PsrR1* ^59^ peaked in fraction 7 (F7) together with the bulk of mRNAs, but there were secondary peaks in mRNA abundance in other fractions (for details, see Riediger et al.^190^). Several proteins and RNA‐protein complexes involved in RNA metabolism, such as RNase D (D), RNase J (J), RNase E (E), RNase P (P), PNPase, enolase (Eno), and CrhR occur in the higher molecular fractions, indicating their association with larger complexes. Most RNAs were detected in overlapping fractions as well, indicating their likely direct association with such complexes. The striking overlap in the in‐gradient distribution of PNPase, enolase, RNase E and J, consistent with their possible colocalization into degradosomes is boxed. Two different gene products were detected for RNase II/R (II/R) and RNase III (III) in the lighter fractions, while Mini‐III was not detected at all. The strong correlation between RNase Z (Z) and the bulk of tRNAs is consistent with the role of this enzyme in tRNA maturation. Note that Hfq was found only in very light fractions, consistent with its non‐RNA binding character in cyanobacteria. Candidates for alternative RNA chaperones are the KhpA/B homologs Slr0287 and Slr1472. See Table 1 for the gene IDs of all other mentioned proteins. The entire data set is available at https://sunshine.biologie.uni-freiburg.de/GradSeqExplorer/. Reprinted in modified form with permission from Riediger et al.^190^.

Gradient profiling by sequencing (Grad‐seq) analyses are an approach to identify unknown RNA chaperones. Following fractionation of whole‐cell lysates on sucrose density gradients by ultracentrifugation, colocalizing proteins and RNA molecules are determined by mass spectrometry and RNA sequencing^177^. This approach led to the discovery of ProQ as a novel major RNA chaperone in *Salmonella enterica* ^177^ and the involvement of exoribonuclease in the stabilization and activation of sRNAs in the gram‐positive pathogen *Streptococcus pneumonia* ^189^. For cyanobacteria, the first such global analysis of stable RNA‐protein complexes has been presented recently, focusing on *Synechocystis* PCC 6803^190^. The stability of such complexes during cell lysis and fractionation was inferred from the colocalization of known RNA‐protein complexes involved in transcription, RNA metabolism, and translation initiation (Figure 4). The data showed the occurrence of a larger number of RNAs in the fractions containing higher molecular weight complexes, suggesting their binding to cognate proteins (Group 1 and Group 2 RNAs in Figure 4). Following hierarchical clustering, the prediction of RNA‐binding protein candidates using RNApred^191^ and considering phylogenetic conservation in 57 and synteny in 34 diverse cyanobacteria, a short list of previously uncharacterized protein candidates for the interaction with sRNAs was obtained^190^. Among them were cyanobacterial homologs of KhpA/B proteins, which are also considered as sRNA chaperones in other bacteria^192^.

## RNA DEGRADOSOME

As the degradation of most RNA species involves several steps catalyzed by different ribonucleases, the rapid turnover of cellular RNAs requires the highly coordinated actions of various ribonucleases^5^. The formation of the RNA degradosome is one of the most important mechanisms for the coordinated actions of RNases found in bacteria. As mentioned earlier, the RNA degradosome of *E. coli* is mainly composed of RNase E, PNPase, RhlB, and enolase (Figure 2B). RNase E as the core of the RNA degradosome recruits other degradosomal components via its C‐terminal noncatalytic region^194^. Another RNase E‐based degradosome identified in the α‐proteobacterium *C. crescentus* is similar to that of *E. coli*, consisting of RNase E, PNPase, a DEAD‐box helicase, and the Krebs cycle enzyme aconitase^17^. In *B. subtilis*, the RNA degradosome is likely composed of RNase Y, RNase J1, RNase J2, and some other proteins^14^. The orthologs of RNase E are universally present in cyanobacteria, while the orthologs of RNase Y are absent (Table 1). The noncatalytic regions of cyanobacterial RNase E proteins are much shorter than those of *E. coli* RNase E, and their primary sequences show no detectable similarity, making it impossible to predict whether an *E. coli*‐like RNA degradosome also exists in cyanobacteria. However, recent works have clearly shown that an RNase E‐based RNA degradosome is present in cyanobacteria (Figure 2A). PNPase was the first to be copurified with the noncatalytic region of RNase E from *Anabaena* PCC 7120 cells, and it interacts with a highly conserved nonapeptide at the very end of the C‐terminus of RNase E within subregion C4^35^. A further copurification assay using the full‐length RNase E identified another RNase E‐associated ribonuclease, RNase II^44^. Fluorescence resonance energy transfer (FRET) analysis using a strain in which RNase E and RNase II were tagged with BFP and CFP respectively revealed that RNase E and RNase II form a compact complex *in vivo* ^44^. *In vitro* assays showed that RNase E binds to RNase II at a stoichiometry of 1:1 via the catalytic region^44^. As RNase E interacts with PNPase and RNase II via its noncatalytic region and catalytic region, respectively, the three ribonucleases are likely to form one complex *in vivo*. Note that the RNase E‐based RNA degradosomes of other bacteria only have one exoribonuclease, PNPase; the benefit of two exoribonucleases coexisting in the degradosome remains unclear. The RNA helicase CrhB was recently shown to interact with the catalytic region of RNase E, suggesting that it might be another component of the cyanobacterial RNA degradosome^172^. The same study also showed that the enolase also interacted with the catalytic region of RNase E, but such an interaction needs to be supported by more evidence.

Taken together, these discoveries clearly show that an RNase E‐based RNA degradosome exists in *Anabaena* PCC 7120. It is very likely that similar RNA degradosomes also exist in other cyanobacteria, as RNase E (particularly, the catalytic region and the PNPase‐interacting motif), PNPase, and RNase II are all highly conserved in cyanobacteria, and CrhB orthologs are also present in most cyanobacterial species. Moreover, these proteins showed overlapping fractionation patterns in the Grad‐seq analysis of *Synechocystis* PCC 6803 (Figure 4). Note that cyanobacterial RNase E has several other conserved motifs of unknown function in the noncatalytic region, and it is possible that the functions of these motifs may be related to interactions with some other unknown degradosomal components or with RNA substrates.

The RNA degradosomes from *E. coli*, *B. subtilis*, and *Anabaena* all contain at least one endoribonuclease, one exoribonuclease, and an RNA helicase, indicating that the concerted actions of these ribonucleases and the helicase are important for the efficient turnover of cellular RNAs. PNPase is present in all known RNase E‐based RNA degradosomes and the PNPase‐binding sites in the RNase E proteins of *E. coli*, *C. crescentus,* and *Anabaena* PCC 7120 have been determined^17^, ^35^, ^195^. Despite the fact that these sites are quite divergent in their amino acid sequences, they are all located at the very end of RNase E, implying that the interaction between PNPase and the very end of RNase E is required for efficient cooperation between PNPase and RNase E.

In different organisms, the importance of RNA degradosome may vary significantly. Deletion of the noncatalytic region of *E. coli* RNase E, which is the scaffold for degradosome assembly, did not lose viability under lab conditions, although it significantly increased the stability of cellular mRNAs^9^. Additionally, among all *E. coli* degradosomal components, only RNase E is indispensable^10^, ^151^, ^196^, ^197^, ^198^. The deletion of RhlB or PNPase only influences the stability of certain RNA species^10^. In *B. subtilis*, all the ribonucleases in the degradosome are dispensable, although the deletion of RNase Y or RNase J1 severely impairs cell growth^16^, ^151^, ^199^. Among the known degradosomal components in cyanobacteria, RNase E is essential for cell viability^47^, ^48^, a characteristic similar to that of *E. coli*. PNPase could not be inactivated in *Synechocystis* PCC 6803 and *Synechococcus* PCC 7002^47^, ^130^, and our attempt to inactivate the PNPase‐encoding gene in *Anabaena* PCC 7120 also failed (unpublished), indicating that PNPase is essential in cyanobacteria. This result contrasts with the finding that deletion of *E. coli pnp* only slightly affects cell growth^148^, ^200^. The importance of RNase II and CrhB in cyanobacteria remains unclear, but they may be less important than RNase E and PNPase, as their orthologs were shown to be dispensable in some unicellular cyanobacteria^130^, ^159^.

## PERSPECTIVES

More than 10 RNA turnover‐related enzymes, including ribonucleases and RNA helicases, have been identified in cyanobacteria by genome analysis. Cyanobacteria are evolutionarily distant from *E. coli* and *B. subtilis* ^21^. Because both *E. coli* and *B. subtilis* have some species‐specific ribonucleases, it is anticipated that cyanobacteria also have such unique enzymes that will need to be discovered experimentally. According to the general principle of RNA degradation in bacteria, the enzymes for at least two activities remain to be identified in cyanobacteria: oligoribonuclease and RNA pyrophosphohydrolase.

**Oligoribonuclease.** Bacterial RNA degradation starts with internal cleavage, and the generated intermediates are further degraded by exoribonucleolytic enzymes from either the 3ʹ or 5ʹ end. However, most exoribonucleases are not able to fully degrade the substrates as their activities can only turn the RNA fragments into oligoribonucleotides of 2–5 nt. These oligoribonucleotides, also known as nanoRNAs, are toxic at least due to their ability to alter global gene expression by priming transcription initiation^201^. Therefore, these oligoribonucleotides need to be converted into single ribonucleotides by oligoribonucleases. The first bacterial oligoribonuclease, Orn, was discovered in *E. coli* ^202^. Orn is required for cell viability, and its depletion leads to a dramatic accumulation of oligoribonucleotides with a length of 2–5 nt^203^. *B. subtilis* has no Orn homologs; however, it contains two distinct oligoribonucleases (NrnA and NrnB) that can complement the *E. coli orn* mutant well^204^, ^205^. The homologs of all these known oligoribonucleases are missing in cyanobacteria, indicating that cyanobacteria may use a different type of enzyme for the degradation of oligoribonucleotides.

**RNA pyrophosphohydrolase.** Bacterial primary transcripts, which have a triphosphate 5ʹ end, are not efficiently recognized by decay‐initiating enzymes, such as *E. coli* RNase E and *B. subtilis* RNase Y. Therefore, these RNAs generally need to be converted into the 5ʹ monophosphorylated form before degradation. This conversion requires the activity of RNA pyrophosphohydrolase. The first RNA pyrophosphohydrolase, RppH, was discovered in *E. coli* ^206^. RppH belongs to the Nudix hydrolase family protein. *B. subtilis* has no close homologs of *E. coli* RppH, but its genome encodes several proteins of the Nudix hydrolase family. One of the Nudix hydrolases, YtkD, was found to have the RNA pyrophosphohydrolase activity and hence was reannotated as *B. subtilis* RppH (BsRppH)^207^. The close homologs of *E. coli* RppH and *B. subtilis* RppH are all absent in cyanobacteria. However, each sequenced cyanobacterium has multiple Nudix hydrolase family proteins, and it is worth testing whether some of them have RNA pyrophosphohydrolase activity.

Although ribonucleases of the same type from different bacteria usually have similar catalytic properties, their cellular substrates are not the same; thus, they can have very different impacts on the growth and physiology *in vivo*. For instance, PNPase is not essential and only important for the growth of *E. coli* at lower temperatures^148^, it is however obligately required for cell viability under normal conditions in cyanobacteria^47^, ^130^. To understand how ribonucleases function in cyanobacteria, it is necessary to investigate the phenotypes of their mutation (or conditional mutation) and overexpression strains and to determine their cellular substrates.

Cyanobacteria have evolved a unique set of RNA‐degrading enzymes. Our understanding of how these enzymes function in cyanobacteria is still very preliminary. In addition, cyanobacteria may contain proteins that regulate the activities of RNA degradation enzymes. These regulatory proteins, such as RraA and RraB, have been found to have important functions in *E. coli* ^208^, ^209^. Continuing to uncover their regulation, cellular substrates, physiological functions, and cooperation mechanisms will help us develop a better understanding of RNA metabolism in cyanobacteria.
